# The Impact of Hyposalivation on Quality of Life (QoL) and Oral Health in the Aging Population of Al Madinah Al Munawarrah

**DOI:** 10.3390/ijerph14040445

**Published:** 2017-04-20

**Authors:** Mohammad S. Ahmad, Ahmed Bhayat, Muhammad Sohail Zafar, Khalid H. Al-Samadani

**Affiliations:** 1 Dental Public Heath, Department of Preventive Dental Sciences, College of Dentistry, Taibah University, Al Madinah Al Munawarrah 41311, Saudi Arabia; msahmad@taibahu.edu.sa; 2 Department of Community Dentistry, School of Dentistry, University of Pretoria, Pretoria 0002, South Africa; ahmed.bhayat@up.ac.za; 3 Department of Restorative Dental Sciences, College of Dentistry, Taibah University, Al Madinah Al Munawarrah 41311, Saudi Arabia; MZAFAR@taibahu.edu.sa; 4 Department of Dental Materials, Islamic International Dental College, Riphah International University, Islamabad 44000, Pakistan

**Keywords:** edentulous, geriatric, hypo salivation, Oral Health Impact Profile-14, xerostomia

## Abstract

Hyposalivation (HS) affects aging individuals by causing pain and discomfort in the oral cavity. The aim here was to determine the impact of hyposalivation and the saliva pH on the quality of life and caries status of geriatrics population. A total of 138 male outpatients attending the Taibah University College of Dentistry (TUCoD) dental clinic were included in the study. The saliva flow, pH, Quality of Life (QoL), and caries status were recorded. The QoL was measured using the Arabic version of the Oral Health Impact Profile-14 (OHIP-14), and the caries status was recorded using the Decayed, Missed, Filled Teeth (DMFT) index. The mean age was 67.5 years and 64% were classified as having hyposalivation. The older respondents tended to have a lower saliva flow and pH compared to their younger counterparts. There was a significant inverse association (*p* = 0.02) between the caries status and mean saliva flow rate. There was also a significant (*p* < 0.001) positive correlation between caries and the OHIP-14 scores (Spearman’s ρ = 0.293). The prevalence of hyposalivation was relatively high and there was an inverse relationship between the age, the saliva flow, and pH. Those with more caries reported significantly poor QoL.

## 1. Introduction

The geriatric population represents an increasing segment of the global population particularly in developed countries [1]. Due to improvement in health care facilities, the average life and life expectancy has increased in recent years [2,3]. For instance, the number of geriatrics (60 years and older) in Saudi Arabia has also increased over the past few years and this has been attributed to the improved quality of life, the improved standard of living, and greater access to health care [1]. The health of elderly individuals may be compromised by conditions such as hypertension, arthritis, stroke, diabetes, and hypercholesterolemia and dry mouth (hyposalivation) [4]. The prevalence of hyposalivation (HS) in people 60 years and older varies from 18% to 65% depending on the sample, geographic location, and the criteria used to define it [4,5]. The salivary flow rate is dynamic and can be affected by multiple factors such as aging, stress, and medications [6,7]. The key factors leading to HS are salivary glands atrophy, dehydration, systemic conditions, and related medications [5,8,9,10]. HS is diagnosed using the saliva flow rate measurement (sialometry) and/or clinical assessments. The use of the saliva flow rate to diagnose HS has been recommended by previous researchers as it has proven to be more reliable and objective compared to clinical assessments and questionnaires [11]. The current study used the saliva flow rate as a marker to diagnose HS in the sample population.

The HS causes difficulty in performing the physiological functions such as speaking, tasting, eating, and swallowing [5,12]. In addition, the lack of therapeutic salivary components (antimicrobial peptides) may affect the localized immunity [13,14]. In addition, the immunoglobulin A (IgA) is another immunological component secreted in saliva that affects the susceptibility of oral and respiratory mucosal infections [15,16]. Therefore, the absence or reduction of IgA may be associated with mucosal host resistance [17]. All of these factors contribute negatively to the oral health-related quality of life in the elderly. The professional dental care can significantly enhance the quality of life for patients by effectively diagnosing and managing the conditions [18,19]. Hence, the prevalence studies are important to report the frequency of HS among the particular population. There has been no study carried out on the prevalence of HS and the impact it has on the oral health related quality of life among geriatrics in the Saudi Arabia. Saudi Arabia has a very hot and dry climate throughout most of the year, which often leads to dehydration and a subsequent increase in HS especially amongst geriatrics. The objectives of this study are to determine the impact of hyposalivation and salivary pH on the quality of life and caries status of the geriatric population.

## 2. Materials and Methods

### 2.1. Study Design

This is a cross-sectional analytical study that was carried out on geriatric dental patients. The study protocol followed the declaration of Helsinki that was reviewed and approved by the ethical research committee of Taibah University, Madinah, Saudi Arabia. All patients older than 60 years attending the outpatient dental clinics (College of Dentistry, Taibah University) were invited to participate in the study. Predefined inclusion and exclusion criteria (Table 1) were used; any patients having any conditions directly affecting the salivary gland tissues such as cancerous lesions or undergoing radiotherapy, chemotherapy were excluded. Patients (60 years and above), ambulatory and mentally stable, were included in the study. This study involved use of cognitive skills (such as spitting for saliva collection) hence the good mental state of patient was considered for inclusion criteria. The mental health of the potential recruits was assessed through verbal communication. The individuals capable to understand and follow the given instructions without any assistance were considered suitable.

A total of 166 patients fulfilling the selection criteria agreed to participate in this study. Each prospective participant was briefed about the proposed protocol and an informed consent was obtained. Three qualified dentists interviewed participants and collected all the necessary data including the medical and dental history, the Quality of Life (QoL) scores (Table 2), and the dental caries (tooth decay) status followed by the collection of the saliva samples for further interpretation. For the ease of understanding and data interpretation, participants were classified into three groups based on the age. 

### 2.2. Patient’s Data Collection

The QoL was measured using the Arabic version of the Oral Health Impact Profile-14 (OHIP-14) questionnaire [20]. The OHIP-14 is a well-accepted and validated research tool that has been used widely for the evaluation of quality of life in relation to oral health [21,22,23]. This self-administrated questionnaire based on seven domains (functional limitation, physical pain, psychological discomfort, physical, psychological, and social disabilities, and handicapped), and 14 key questions were used (Table 2). All participants were asked to rate each question using a Likert scale. The Likert scale is commonly used to record participant’s response during the questionnaire-based studies [24,25]. The Likert scale can range from never (Score 0), hardly ever (Score 1), occasionally (Score 2), fairly often (Score 3), very often (Score 4), to always (Score 5). The total scores were calculated by adding the scores for each domain. The scores can range from 0 to 70, and a score above 10 indicates a poorer QoL. 

The status of tooth decay (dental caries) was measured using the Decayed, Missing, Filled Teeth (DMFT) index. The DMFT is an authentic and the most commonly used index for epidemiological assessment of dental caries [26,27,28]. Briefly, in this index, teeth are coded as carious (decayed) only if there is cavitation or undermined enamel. “Missing” refers to teeth extracted due to caries, i.e., the patient reports a history of cavitation. Any teeth having restoration or filling are counted as filled teeth. The clinical examinations were carried out on the dental chair equipped with dental light and according to the World Health Organization (WHO) criteria [29]. The dentist was blinded during and after clinical examination with regard to patient’s saliva flow rate and the pH. The collective DMFT scores were categorized into four groups: low caries (DMFT Scores 0 to 10), moderate caries (DMFT Scores 11 to 20), and high caries (DMFT Scores 21 to 31), and DMFT scores of 32 (maximum) means either all teeth are missing, decayed, or both (i.e., no health tooth).

### 2.3. Saliva Sampling and Measurement of Saliva Flow Rate and pH

Another dentist, who was blinded to the patient’s caries status, medical and dental history, and clinical examination, collected the saliva samples. The unstimulated saliva was collected in the morning (8:00–10:00 am; Arabia Standard Time, AST) by asking the participants to rinse out their mouth and then spit into a container for two minutes. All participants were observed during this process to make sure the complete spitting of whole mouth saliva secreted in the given time regardless of number of spits. The volume of the saliva was measured using a syringe to determine the saliva flow rate in mL/min. All participants with a saliva flow rate less than or equal to 0.9 mL/min were classified as having a low flow rate (hyposalivation). Although variable criteria for defining hyposalivation have been used, the criteria used in this study (0.9 mL/min) was defined following the previous studies [30,31]. In addition, the physiological salivary flow rate of 1 mL/min or greater has been classified as normal [32]. Hence, a salivary flow rate less than physiological (1 mL/min) has been considered as hyposalivation. The pH of the saliva was recorded using saliva pH strips (Simplex Health, Wellingborough, UK). The saliva pH strips was placed into the saliva container and pH value was recorded for each specimen according to the manufacturer’s instructions. The pH was classified into two groups, either low (pH ≤ 6.9) or normal (pH ≥ 7.0).

### 2.4. Statistical Analysis

All data was entered and analyzed using the statistical package for social science IBM SPSS, version 21 (IBM, Armonk, NY, USA). The characters of variables were described using frequency distribution, means, and standard deviation (SD) for continuous variables. The analysis of variance (ANOVA) and Kruskal–Wallis tests were used to determine the association between variables. The saliva samples were coded to maintain anonymity but to also allow it to be linked to the participants’ caries status.

### 2.5. Ethical Statement

All procedures performed in studies involving human participants were accordance with the ethical standards of the institutional and/or national research committee and with the 1964 Helsinki declaration and its later amendments or comparable ethical standards (TUCODREC:11032015). An informed consent was obtained from all individual participants included in the study.

## 3. Results

Among 166 patients, a total of 138 (83%) agreed to participate in the study. The majority of patients was excluded due to their disengagement, lack of cooperation during the saliva collection, and/or did not complete the questionnaires. The mean age was 67.5 ± 7.4 years, and almost two-thirds (64%) were diagnosed for hyposalivation as their saliva flow rate being 0.9 mL/min or less. The OHIP-14 questionnaire proved to have a good internal consistency (Cronbach α= 0.76) and correlation between the domains and total scores (0.64 to 0.79). Table 3 shows the association between the mean saliva flow rate and the pH in relation to the age and caries status of the participants.

There was an inverse relationship between the age and the saliva flow rate and pH. With aging, the flow and pH of the saliva was decreased. Similarly, there was a significant association between the caries status and the saliva flow rate. As the saliva flow decreased, the prevalence of caries increased and those with the slowest mean saliva flow rate were completely edentulous. The total mean OHIP-14 score was 6.09 (±7.69) and ranged from 0 to 43. The mean score, SD, and range for each domain are shown in Table 4. The highest scores (1.91 ± 1.69) were recorded for the physical pain followed by functional limitation (0.90 ± 1.54) and physical disability (0.88 ± 1.62). In contrast, the lowest mean scores were recorded for social disability (0.55 ± 1.07) and feeling handicapped (0.43 ± 1.18). 

There was a significant positive correlation between the DMFT and the OHIP-14 scores (*p* < 0.001; Spearman’s ρ = 0.293). For instance, an increase in the DMFT scores led to an increase in OHIP scores and vice versa. In order to confirm this correlation, the mean of OHIP-14 domain total scores and the severity of dental caries have been compared. The total mean OHIP-14 score and almost all the mean domain scores increased significantly as the DMFT increased for instance in the case of high DMFT (Table 5). Hence, the positive correlation between the OHIP-14 scores and the DMFT status was validated. The edentulous cohort had significantly higher scores for all domains, and a mean score of more than double for the total OHIP-14. Almost two-thirds (64%) of the sample had a saliva flow rate (≤0.9 mL/min) lower than normal. Although there were no significant differences in the domain scores, all those with a low flow rate scored considerably higher mean scores compared to the participants with a normal flow rate (1 mL/min or more).

In terms of correlating the individual OHIP domain and salivary flow rates, the handicapped domain was the only domain that showed a normal flow rate scored higher (0.51 ± 1.50) compared to those with a low saliva flow rate (0.38 ± 0.97). The mean scores for rest of all six OHIP domains (functional limitation, physical pain, psychological discomfort, physical disability, psychological disability, and social disability) remain higher for a low saliva rate compared to a normal saliva flow rate. The mean scores for each domain is shown in Table 6. Furthermore, participants with a low flow rate had a significantly (*p* = 0.008) higher mean DMFT score (18.94 ± 10.42) compared to the DMFT score (14.14 ± 9.10) of those with a normal flow rate (Table 6). 

Interestingly, only one-third of the participants had a neutral pH (7.0) or greater. Those with a pH score less than 7 reported a poorer quality of life compared to those with a higher pH value Table 7. The OHIP-14 scores of various domains have been compared in relation to lower and normal pH (Table 7). All OHIP-14 domains scores remain insignificantly higher in case of low pH compared to normal pH. The only exception is the social disability domain.

There was a significant difference (*p* = 0.02) in the mean scores for the social disability domain; those with lower pH scores reported feeling more socially disabled compared to their counterparts. Although not statistically significant, those diagnosed with a low pH (≤6.9) tended to have a higher mean DMFT score compared to those with a high pH (≥7.0).

## 4. Discussion

The current study was conducted to evaluate the prevalence of hyposalivation (HS) and its relationship with the saliva pH and quality of life in the aging population. The reduced salivary flow is a commonly seen in the aging populations. This can be attributed to either age-related localized degeneration of salivary glands [33,34,35] or systemic diseases [33,36]. The prevalence of such conditions may vary among populations. For instance, the prevalence of HS (64%) has been much higher than reported (30%) in Americans [37]. It was, however, closer to the 56% reported in a study done in Finland [8]. The relatively high prevalence has been reported in the current study that could be attributed to the fact that the current cohort resides in a much hotter and drier climate compared to the sample from other studies [8,37]. This increased heat and lack of humidity could be a contributing factor for the increased prevalence of xerostomia. The increased environmental temperature (such as heatwaves), warmer indoor rooms, or higher body temperature (fever) result in dehydration of body and dryness of mucosa, hence requiring additional intake of fluids to rehydrate the mucosa [38]. In addition, the superior access and better utilization of dental services in the developed countries contributed to the reduction in the prevalence of HS [37,39]. Lastly, the other studies derived their sample population from nursing homes or old age care facilities; the current study has been conducted at an institutional dental clinic. Patients who attend the dental clinic would have more dental problems and possibly more HS compared to patients who do not attend dental facilities. Therefore, this sample could have overestimated the actual prevalence of HS in Madinah. It is, however, useful for oral health care practitioners to be aware of the potential prevalence of HS as it does have implications for the management and treatment planning. 

Similar to other studies [37,39], there was an inverse association between the age and saliva flow rates. As the patient ages, the organs atrophy and often result in a decrease in output function. The major salivary glands (parotid) have shown age-related atrophy changes, possibly due to the reduced flow rate in older cohorts [12,39]. The mean OHIP-14 score was 6.09 (±7.69) and ranged between 0 and 43. This was considerably higher than the mean of 3.0 and 3.4 reported in other studies [21,40]. The edentulous cohort had a mean score of 10.09, which was significantly higher than their dentate counterparts. This confirms the results of a previous study [41] that has highlighted the importance of retaining the natural dentition, and confirms the value and importance of natural teeth and its positive effects on the quality of life in geriatrics [41].

Possible reasons for the high mean score reported in the current study could be attributed to the development and socioeconomic status of the participants and the country in which this study was undertaken. The other studies [42,43] were conducted in developed countries offering good quality dental care more frequently and better access to the dental services. By having more opportunities to visit oral health professionals, it could mean that their dental problems were promptly diagnosed, managed, and treated earlier on. Such earlier diagnosis and dental treatment is likely to reduce the DMFT score significantly and improve their quality of life [42]. However, similar to these studies, the highest mean score was reported for physical pain. This meant that pain was common in elderly patients, and this could be the reason for them attending the dental clinics. The handicapped domain scored the lowest mean score and meant that, although participants were in pain, they either did not feel handicapped or did not like to express such feelings. This could be due to having dentures or sufficient teeth for to chew their food properly.

The current study reported a significant (*p* < 0.001) positive correlation between the DMFT and the OHIP-14 scores (Spearman’s ρ = 0.293). There was also a significant association between the categories of the DMFT and the mean OHIP-14 scores. As the dental caries got worse, it impacted on the quality of life and as a result there was a significant increase in the mean domain and total scores. This is similar to many other studies, which stressed the importance of good oral health to ensure good mental, social, and physical well-being [43,44]. However, this result was in contrast to that reported in a Norwegian study, which found no association between caries and the quality of life [40]. This lack of an association between the variables could be attributed to the improved access to dental services and the low caries prevalence in the Norwegian population [40].

Almost two-thirds (64%) of the sample had a low salivary flow rate, and this could be attributed to their age as reported in other studies [4]. Those with lower domain scores generally had a lower saliva flow rate. This showed the impact that the dry mouth has on these individuals as it affects all aspects of their quality of life. Participants with low salivary flow rate also had significantly more dental caries compared to those with a higher saliva flow rate. This confirms the results of other studies, including a systematic review that reported a strong association between saliva flow and dental caries [45]. In addition to salivary flow rate, there are other factors that are involved in inhibiting the cariogenic microbial flora. For example, there are certain antimicrobial peptides present in the saliva [46,47,48] that play their role against cariogenic bacteria. In addition, saliva acts as a buffer and a mechanical cleanser and physically removes bacterial plaque, bacterial metabolites, and regulates the pH. These factors are likely to reduce the prevalence of caries [45]. As a result of reduced salivary flow, these functional components of saliva are also compromised, hence enhancing the likeliness of tooth decay. 

Similar to a low salivary flow rate, the low pH scores reported a poorer quality of life compared to those with a normal pH value. This can be explained by the burning sensation experienced by many elderly patients who have been diagnosed with HS. The low pH would increase the burning sensation, and this could cause the low quality of life scores as reported. Those with a low pH score also had a higher mean caries score compared to those with a normal pH [8,12]. This could be due to the acidic environment that would lead to enamel demineralization [49,50]. The acidic pH results in the outflow of minerals that leads to weakening of enamel and cavitation [51]. In terms of dental restorations, fluoride releasing materials such as glass ionomers and giomers are recommended for filling the decayed teeth [52,53,54]. Considering that the decreased salivary flow and pH are associated with dental caries, patients may hence suffer from the symptoms of dental caries (such as pain, hypersensitivity), affecting the quality of life. 

There are a few limitations to the current study. Although this is a cross-sectional study that represented a data of sample from a geriatric population, it does provide useful baseline data for planning, training, and managing patients suffering from decrease salivary flow rate. The benefits of sterile saliva collection devices have recently been reported [55] and can be used for future studies. The etiology of HS was not taken into consideration, as this study focused only on the prevalence and its association with dental caries and quality of life. Future studies have been initiated to identify the confounding effects of medications on the saliva flow, pH, and dental caries.

## 5. Conclusions

There was an inverse relationship between the age, saliva flow, and pH. As the age increases, the flow rate and pH of the saliva decreases. Both hyposalivation and low salivary pH are the contributory factors for a poorer quality of life. In addition, higher caries prevalence has been observed compared to those with a normal salivary flow rate. In addition, high caries prevalence has been reported to be associated with significantly poorer quality of life compared to low caries prevalence. 

## Figures and Tables

**Table 1:** The selection criteria used for recruiting patients for this study.

Inclusion Criteria	Exclusion Criteria
Patients older than 60 years	Cancerous lesion in head and neck region
Good state of mental health	History of chemotherapy or radiotherapy
No swelling or tenderness of major salivary glands including parotid, submandibular and sublingual	Surgical excision of any major salivary glands
	Autoimmune diseases affecting salivary glands such as Sjögren syndrome

**Table 2:** The description of Quality of Life (QoL) questionnaire; Oral Health Impact Profile-14 (OHIP-14) criteria questions and domains used in this study.

Oral Health Impact Profile-14 Criteria for QoL Assessment (14 Questions from 7 Domains)		
Questions regarding oral tissues/dentures	Domain	Likert score rated by patient
Problem in pronouncing words	Functional limitation	0__1__2__3__4__5__
Altered sense of taste		0__1__2__3__4__5__
Difficulty in chewing	Physical pain	0__1__2__3__4__5__
Pain/aching		0__1__2__3__4__5__
Worry about dental problems	Psychological discomfort	0__1__2__3__4__5__
Psychological discomfort		0__1__2__3__4__5__
Problem affecting the diet	Physical disabilities	0__1__2__3__4__5__
Interruption in meals		0__1__2__3__4__5__
Difficult to relax	Psychological disabilities	0__1__2__3__4__5__
Feeling embarrassed		0__1__2__3__4__5__
Feeling irritable from others	Social disabilities	0__1__2__3__4__5__
Job related difficulties		0__1__2__3__4__5__
Least satisfied in life	Handicapped	0__1__2__3__4__5__
Functional inability		0__1__2__3__4__5__

Likert scale (0–5; 0: never, 5: always).

**Table 3:** Mean saliva flow rate and pH of saliva in relation to age and Decayed, Missed, Filled Teeth (DMFT) categories (*N* = 138).

Age	Saliva Mean Flow Rate in mL/min (±SD)	Saliva Mean pH (±SD)
60–65 (*n* = 82)	0.78 (±0.27)	6.82 (±0.52)
66–70 (*n* = 29)	0.75 (±0.30)	6.83 (±0.38)
≥71 (*n* = 27)	0.71 (±0.20)	6.61 (±0.56)
* *p*-Value	0.49	0.21
**DMFT**	**Saliva Mean Flow Rate in mL/min (±SD)**	**Saliva Mean pH (±SD)**
Low (*n* = 37)	0.87 (±0.28)	6.83 (±0.39)
Moderate (*n* = 51)	0.76 (±0.26)	6.64 (±0.61)
High (*n* = 15)	0.73 (±0.20)	7.03 (±0.13)
Edentulous (*n* = 35)	0.66 (±0.25)	6.83 (±0.51)
* *p*-Value	0.02	0.06
Total (*N* = 138)	0.76 (±0.27)	6.78 (±0.51)

* *p*-Value calculated using the Kruskal–Wallis test. SD: standard deviation.

**Table 4:** Mean and range values for each domain and the total OHIP-14 scores (*N* = 138).

OHIP-14 Domains	Mean Scores (±SD)	Range
Functional limitation	0.90 (±1.54)	0–8
Physical pain	1.91 (±1.69)	0–6
Psychological discomfort	0.66 (±1.49)	0–8
Physical disability	0.88 (±1.62)	0–8
Psychological disability	0.77 (±1.44)	0–6
Social disability	0.55 (±1.07)	0–5
Handicapped	0.43 (±1.18)	0–8
Total OHIP14 score	6.09 (±7.69)	0–43

**Table 5:** Association between the DMFT categories and the mean domain and total scores (mean (±SD)) for the OHIP-14.

DMFT	FL	PP	PsDc	PhD	PsDa	SoD	Hd	Total
Low (*n* = 37)	0.27 (±0.84)	1.57 (±1.56)	0.32 (±1.45)	0.68 (±1.47)	0.43 (±1.24)	0.35 (±0.82)	0.65 (±1.70)	4.27 (±7.39)
Moderate (*n* = 51)	0.73 (±1.30)	1.47 (±1.36)	0.47 (±0.92)	0.63 (±1.30)	0.80 (±1.66)	0.39 (±0.83)	0.24 (±0.71)	4.73 (±5.69)
High (*n* = 15)	0.47 (±1.06)	2.53 (±1.60)	0.60 (±1.06)	0.00 (±0.00)	0.40 (±1.12)	0.20 (±0.41)	0.20 (±0.78)	4.40 (±3.62)
Edentulous (*n* = 35)	2.00 (±2.03)	2.63 (±2.00)	1.31 (±2.08)	1.83 (±2.09)	1.23 (±1.31)	1.14 (±1.52)	0.57 (±1.20)	10.71 (±9.88)
*p*-Value *	<0.001	0.010	0.001	<0.001	0.001	0.002	0.273	<0.001

* *p*-Value calculated using the Kruskal–Wallis test; Various domains of OHIP-14: FL = Functional limitation; PP = physical pain; PsDc = psychological discomfort; PhD = physical disability; PsDa = psychological disability; SoD = social disability; HD = handicapped.

**Table 6:** Association between mean OHIP-14 scores, caries status, and saliva flow rate (*N* = 138).

OHIP-14 Domain Mean Scores (±SD)	Saliva Flow Rate (mL/min)		
	Low (*n* = 89)	Normal (*n* = 49)	* *p*-Value
Functional limitation	1.04 (±1.36)	0.63 (±1.81)	0.13
Physical pain	1.92 (±1.55)	1.88 (±1.92)	0.89
Psycho discomfort	0.67 (±1.16)	0.63 (±1.97)	0.88
Physical disability	0.93 (±1.37)	0.78 (±2.00)	0.59
Psycho disability	0.82 (±1.45)	0.67 (±1.42)	0.57
Social disability	0.61 (±0.10)	0.45 (±1.19)	0.41
Handicapped	0.38 (±0.97)	0.51 (±1.50)	0.54
OHIP-14	6.38 (±5.86)	5.55 (±10.26)	0.55
DMFT score	18.94 (±10.42)	14.14 (±9.10)	0.01

* *p*-Value calculated using ANOVA.

**Table 7:** Association between mean OHIP-14 scores, caries status and saliva pH (*N* = 138).

OHIP-14 Domains Mean Scores (±SD)	Saliva pH		* *p*-Value
	Low (≤6.9) (*n* = 93)	Normal (≥7) (*n* = 45)	
Functional limitation	0.94 (±1.67)	0.82 (±1.27)	0.69
Physical pain	1.97 (±1.83)	1.78 (±1.35)	0.54
Psycho discomfort	0.75 (±1.55)	0.47 (±1.34)	0.29
Physical disability	0.90 (±1.68)	0.82 (±1.51)	0.78
Psycho disability	0.89 (±1.54)	0.51 (±1.18)	0.14
Social disability	0.70 (±1.21)	0.24 (±0.61)	0.02
Handicapped	0.43 (±1.19)	0.33 (±1.26)	0.52
OHP-14	6.62 (±7.95)	4.98 (±7.06)	0.24
DMFT score	17.92 (±10.41)	15.82 (±9.72)	0.26

* *p*-Value calculated using ANOVA.
